# Untreated duration predicted the severity of depression at the two-year follow-up point

**DOI:** 10.1371/journal.pone.0185119

**Published:** 2017-09-21

**Authors:** Ching-I Hung, Chia-Yih Liu, Ching-Hui Yang

**Affiliations:** 1 Department of Psychiatry, Chang Gung Memorial Hospital at Linkou and Chang Gung University College of Medicine, Tao-Yuan, Taiwan; 2 Department of Nursing, Chang Gung University of Science and Technology, Tao-Yuan, Taiwan; Chiba Daigaku, JAPAN

## Abstract

**Background:**

No study has investigated the impact of the duration of untreated depression (DUD) on the severity of depression at the two-year follow-up point in patients with major depressive disorder (MDD) who discontinued pharmacotherapy. This study aimed to investigate this issue.

**Methods:**

This study enrolled 155 subjects with MDD at baseline, and 101 subjects who had discontinued pharmacotherapy for 17.1 ± 5.8 months were assessed at the two-year follow-up point. DUD was defined as the interval between the onset of the index major depressive episode and the start of pharmacotherapy. The 17-item Hamilton Depression Rating Scale (HAMD) was used to evaluate depression. Multiple linear regressions were used to examine the impacts of DUD on the severity and improvement percentage (IP) of depression at follow-up.

**Results:**

A longer DUD was significantly associated with a greater severity and a lower IP of depression at follow-up. After controlling for confounding factors, DUD was the most significant factor predicting the severity and IP of depression at follow-up. DUD was more strongly associated with the prognosis of depression at follow-up than depression and anxiety severities at baseline.

**Conclusions:**

The DUD at baseline independently predicted the severity of depression at the two-year follow-up point. Although the patients had discontinued pharmacotherapy for nearly 1.5 years, the impact of the DUD on the severity of depression persisted at follow-up. The DUD was an important index that predicted the severity of depression at the two-year follow-up point.

## Introduction

The duration of untreated episode (DUE) and duration of untreated illness (DUI) have important impacts on the treatment response and prognosis of major depressive disorder (MDD) [1–9]. The DUI for patients with MDD was defined as the interval between the first major depressive episode (MDE) and the start of adequate treatment [1]. The DUE for patients with MDD was defined as the interval between the onset of a recurrent MDE and the start of adequate treatment [1]. The duration of untreated depression (DUD) for the index episode was defined as the interval between the onset of the current MDE and the start of the first adequate treatment; i.e., the DUI for those with a first MDE and the DUE for those with a recurrent MDE [1]. A shorter duration of untreated depression is associated with higher response and remission rates, as well as less disability post-pharmacotherapy [1]. Moreover, a shorter DUE is associated with a faster response to antidepressant treatment [7]. The remission rate gradually decreases with a longer DUI for MDD patients experiencing a first episode, especially if pharmacotherapy is initiated more than six months after onset of the first depressive episode [3,8]. Although several studies have investigated the associations of DUI or DUE with the outcome of MDD [2], the duration from enrollment to the follow-up point in these studies ranged from several months to one year. One previous study reported that DUD was not significantly associated with the time of recovery from a MDE post-pharmacotherapy at the two-year follow-up [10]. However, the major purpose of the study was not focused on investigating the impact of DUD on the outcome of depression at the two-year follow-up point. Moreover, few studies have examined the DUI and DUE simultaneously based on Ghio’s report [1,7]. The impact of the DUD, which includes the DUI and DUE, on the prognosis of MDD at the 2-year follow-up point have been neglected and should be investigated.

Nearly half of patients discontinue antidepressant treatment within 6 months [11], meaning that a proportion of patients with MDD in clinical practice have discontinued pharmacotherapy at the two-year follow-up point. In investigating the impacts of DUD on the prognosis of depression, previous studies have focused on treatment responses or outcome post-pharmacotherapy [2]. The impact of DUD on the outcome of depression among MDD patients who discontinued pharmacotherapy has been neglected. This raises an interesting question: does the impact of DUD on the prognosis of depression still persist at the two-year follow-up point among MDD patients who have discontinued pharmacotherapy? To the best of our knowledge, no study has investigated this issue. Clarification of this issue is important, because it may 1) help physicians to understand the impact of DUD on the prognosis of depression, then alert physicians to monitor this factor; and 2) encourage patients with MDD to accept treatment as soon as possible. Therefore, the purpose of this study was to investigate the impact of DUD on the severity of depression at the 2-year follow-up point, focused on patients with MDD who had discontinued pharmacotherapy. We hypothesized that the impacts of DUD persist, even in patients who have discontinued pharmacotherapy, and that patients with a longer DUD have a poorer prognosis of depression.

## Methods

### Subjects

The study was approved by the Institutional Review Board of the Chang Gung Memorial Hospital, a medical center in northern Taiwan. The study enrolled subjects at baseline, from September 2005 to August 2007, in the psychiatric outpatient clinics of the same hospital. MDD and a MDE were diagnosed based on the Structured Clinical Interview for DSM-IV-text revision (TR) Axis I Disorders [12]. The inclusion criteria were as follows: consecutive outpatients (18 to 65 years of age) who 1) met the criteria for MDD and were in a current MDE [13]; and 2) had not taken antidepressants or other psychotropic drugs for at least the past four weeks. Patients older than 65 years of age were excluded because they may be at increased risk of cerebrovascular diseases and brain degenerative diseases, which could confound the prognosis of depression. Three exclusion criteria were established to prevent the severity of depression being confounded: 1) a history of substance dependence or abuse without full remission in the past month; 2) psychotic symptoms, catatonic features, or severe psychomotor retardation; and 3) hypertension, diabetes mellitus, and other chronic medical diseases. The second exclusion criteria excluded outpatients with a compromised capacity to consent. Therefore, the enrolled subjects with MDD had the capacity to consent. Written informed consent, based on the guidelines regulated in the Declaration of Helsinki, was obtained from all subjects prior to enrollment. The third exclusion criterion was established because comorbidity with some chronic medical diseases might confound the prognosis of depression.

The definition of the DUD has been described in the Introduction. DUD was assessed at baseline. At baseline, a board-certified psychiatrist, who was blind to the severity of depression and other data, interviewed the subjects to assess the onset time point of the index MDE. Moreover, two durations were recorded at the follow-up point, including the total duration of pharmacotherapy in the two-year period and the duration of discontinuance of pharmacotherapy, which was defined as the duration from the time point of discontinuation of pharmacotherapy to the two-year follow-up point.

### Psychometric scales

The 17-item Hamilton Depression Rating Scale (HAMD) was used as the major scale for evaluating the outcome of depression at baseline and at the two-year follow-up point [14]. The Hospital Anxiety and Depression Scale (HADS), which is composed of a 7-item depression subscale (HADS-D) and a 7-item anxiety subscale (HADS-A), was used to evaluate the severities of depression and anxiety at baseline [15]. The HADS-A was used as a variable to control the severity of anxiety at baseline. The total scores ranged from 0 to 52 for the HAMD and 0 to 21 for the HADS-D and HADS-A. A higher score indicated a greater severity.

### Procedures

After enrollment, the HAMD was evaluated and the HADS was administered. The subjects were treated with venlafaxine extended-release, one 75 mg capsule per day, and zolpidem during the first four weeks. After the four-week treatment, pharmacotherapy was not controlled, and the patients were treated as general psychiatric outpatients. Some patients discontinued pharmacotherapy due to improvement or other reasons during the two-year period. These subjects were followed-up at the two-year point. Subjects who met the following three conditions were categorized as the exclusion group: 1) subjects who refused a follow-up assessment; 2) subjects who were unable to be contacted by mail or phone; and 3) subjects who were still undergoing pharmacotherapy at the two-year follow-up point. Only subjects who attended a follow-up appointment and who were not undergoing pharmacotherapy in the index follow-up month were included in the statistical analyses, and were categorized as the inclusion group.

At follow-up, the psychiatric data were evaluated by the same psychiatrist, including the HAMD score and other data. The HAMD score and the improvement percentage (IP) of the HAMD score at follow-up were considered as the major indices by which to measure the outcome of depression. The IP was calculated as (score at baseline—score at the two-year follow-up) / score at baseline.

### Statistical methods

All statistical analyses were performed using SPSS for Windows 20.0 (SPSS Inc., IBM Corporation, Armonk, NY, USA). The independent *t* test, Mann-Whitney U test, paired *t* test, Wilcoxon signed ranked test, Chi-square test, and Spearman’s correlation were used in appropriate situations. Spearman´s correlation and some nonparametric tests were used because the DUD and some parameters did not fulfil the criteria of a normal distribution.

To understand the impacts of DUD on the severity and IP of depression at follow-up, two multiple linear regressions with the forced entry method were performed. In the first and second regression models, the dependent variables were the HAMD score at follow-up and the IP of the HAMD score at follow-up, respectively. Fourteen factors were considered as possible independent variables, including five demographic variables (age, sex, educational years, employment status, and marital status) at baseline, DUD, total duration of pharmacotherapy during the two years, duration of discontinuance of pharmacotherapy, age at first MDE, nature of MDE (i.e., first or recurrent MDE), number of episodes, and three psychometric (HAMD, HADS-D and HADS-A) scores at baseline. However, only variables that were significantly correlated with the dependent variable in the univariate analysis or that had been reported to be important factors related to the DUI or DUE were placed into the regression models. A two-tailed *P* value < 0.05 was considered statistically significant in all statistical analyses.

## Results

### Subjects

At baseline, 155 patients with MDD were enrolled. At the two-year follow-up point, 131 attended a follow-up appointment, 11 refused to participate in a follow-up assessment, and 13 were unable to be contacted. Among the 131 subjects who were followed-up, 30 were undergoing pharmacotherapy in the index follow-up month. The remaining 101 subjects, who had discontinued pharmacotherapy and attended follow-up, were included in the statistical analyses (S1 Dataset).

For clarity, the labels “(B)” and “(2Y)” are used to represent data collected at baseline and the 2-year point, respectively. Table 1 shows the differences in demographic variables, psychometric scores, DUD_(B)_, and other variables between the exclusion and inclusion groups. In the inclusion and exclusion groups, 75 (74.3%) and 43 (79.6%) subjects were suffering a first episode (Table 1), and 26 (25.7%) and 11 (20.4%) had experienced multiple episodes at enrollment, respectively. No significant differences were noted in these variables between the inclusion and exclusion groups. Table 1 also shows the psychometric scores and IP of the HAMD score at follow-up. In the 101 subjects, the total duration of pharmacotherapy and the duration from discontinuation of pharmacotherapy to the follow-up point were (mean ± standard deviation) 5.5 ± 4.5 and 17.1 ± 5.8 months, respectively. Compared with the HAMD, HADS-D and HADS-A scores at baseline, the three psychometric scores exhibited significant decreases at the two-year follow-up point (*P* < 0.001).

**Table 1:** Demographic variables and psychometric scores of subjects in the exclusion and inclusion groups ^a^^,^^b^^,^^c^.

	Exclusion group (*n* = 54)	Inclusion group (*n* = 101)	*P*
Age at enrollment_(B)_ (years)	29.9 ± 7.6	30.4 ± 8.2	0.72
Women_(B)_ (%)	70.4	67.3	0.72
Educational years_(B)_	13.0 ± 2.8	13.6 ± 2.3	0.14
Married_(B)_ (yes, %)	37.0	39.6	0.86
In employment_(B)_ (yes, %)	63.0	57.4	0.61
First episode_(B)_ (yes, %)	79.6	74.3	0.55
Number of episodes	1.24 ± 0.51	1.30 ± 0.56	0.54
Age at first episode_(B)_ (years)	27.0 ± 8.2	27.7 ± 8.6	0.64
Duration of untreated depression_(B)_ (months) ^d^	18.0 ± 25.2	16.3 ± 23.5	0.69
HAMD score_(B)_	23.7 ± 3.8	23.2 ± 4.1	0.42
HADS-depression score_(B)_	14.6 ± 3.6	14.2 ± 3.3	0.44
HADS-anxiety score_(B)_	15.3 ± 3.1	14.5 ± 3.5	0.18
Total duration of pharmacotherapy_(2Y)_ (months)	-	5.5 ± 4.5	-
Duration of discontinuation of pharmacotherapy_(2Y)_ (months)	-	17.1 ± 5.8	-
HAMD score_(2Y)_	-	9.9 ± 7.7	-
HADS-depression score_(2Y)_	-	6.5 ± 5.0	-
HADS-anxiety score_(2Y)_	-	8.5 ± 4.5	-
Improvement percentage of HAMD score_(2Y)_	-	57.7 ± 31.0	-

HAMD = Hamilton Depression Rating Scale; HADS = Hospital Anxiety and Depression Scale.

^a^ (B): Data recorded at baseline; (2Y): Data recorded at the two-year follow-up point.

^b^ There were no significant differences in the baseline data between the two groups.

^c^ Continuous variables are presented as the mean ± standard deviation.

^d^ The first quartile, median, and third quartile were 1.5, 4.0, and 25.5, respectively, for the inclusion group, and 3.1, 8.5, and 39.0, respectively, for the exclusion group.

In the 101 subjects, there was no significant difference in DUD_(B)_ between the male and female subjects, between patients with and without employment, and between patients who were and were not married. The correlations of DUD_(B)_ with age, educational years_(B)_, and HAMD_(B)_ score were not significant.

### Correlations of variables at baseline with the severity and IP of depression at follow-up

Table 2 shows that DUD_(B)_ and the HAMD_(B)_ score were significantly and positively correlated with the HAMD_(2Y)_ score. Educational years_(B)_ was significantly and negatively correlated with the HAMD_(2Y)_ score. DUD_(B)_ was significantly and negatively correlated with the IP of the HAMD_(2Y)_ score. The HAMD_(B)_, HADS-A_(B)_ and HADS-D_(B)_ scores were not significantly correlated with the IP of the HAMD_(2Y)_ score.

**Table 2:** Spearman´s correlations of psychometric scores and improvement percentage of depression at follow-up with variables at baseline ^a^^,^^b^.

	HAMD_(B)_	HAMD_(2Y)_	IP of HAMD_(2Y)_
Duration of untreated depression_(B)_	0.12	0.25*	−0.24*
Age at enrollment_(B)_	0.10	0.10	-0.08
Educational years_(B)_	−0.25*	−0.22*	0.19
HAMD_(B)_	-	0.22*	−0.05
HADS-depression_(B)_	0.36**	−0.07	0.12
HADS-anxiety_(B)_	0.32**	0.08	−0.04
Age at first episode_(B)_	0.02	0.01	0.00
Total duration of pharmacotherapy_(2Y)_	0.04	−0.10	0.09
Duration of discontinuation pharmacotherapy_(2Y)_	−0.08	0.06	-0.06

* *P* < 0.05;

** *P* < 0.01.

HAMD = Hamilton Depression Rating Scale; HADS = Hospital Anxiety and Depression Scale; IP = improvement percentage.

^a^ (B): Data recorded at baseline; (2Y): Data recorded at the two-year follow-up point.

^b^ Table 2 shows the Spearman´s correlation coefficients.

The total duration of pharmacotherapy during the two years and the duration of discontinuation of pharmacotherapy were not significantly correlated with the HAMD_(2Y)_ score or the IP of the HAMD_(2Y)_ score (Table 2).

The correlations of DUD_(B)_ with the HAMD_(2Y)_ score and the IP of the HAMD_(2Y)_ score were significant in subjects (n = 30) undergoing pharmacotherapy in the index follow-up month (Spearman’s correlation coefficients = 0.43 and -0.42, *p* = 0.02 and 0.02, respectively). In all subjects who accepted follow-up (n = 131, including 101 subjects without pharmacotherapy and 30 with pharmacotherapy at follow-up), the correlations of DUD_(B)_ with the HAMD_(2Y)_ score and the IP of the HAMD_(2Y)_ score were significant (correlation coefficients = 0.31 and -0.30, *p* < 0.001 and 0.001, respectively).

### Differences in the severity and IP of depression at follow-up in subjects according to categorical variables

Table 3 shows the variations in HAMD_(2Y)_ score and IP of the HAMD_(2Y)_ score in subjects according to categorical variables. No significant differences in the HAMD_(2Y)_ score or the IP of the HAMD_(2Y)_ score were noted between men and women, between first-episode and multiple-episode subjects, between married and single patients, or between patients with and without employment.

**Table 3:** Severity and improvement percentage of depression in patients according to categorical variables ^a^^,^^b^.

		HAMD_(B)_	HAMD_(2Y)_	IP of HAMD_(2Y)_
Women_(B)_	Yes (*n* = 68)	23.5 ± 4.2	10.0 ± 8.2	57.9 ± 32.3
	No (*n* = 33)	22.5 ± 3.8	9.7 ± 6.6	57.2 ± 28.6
Employment_(B)_	Yes (*n* = 58)	23.2 ± 3.7	9.8 ± 7.8	57.8 ± 32.4
	No (*n* = 43)	23.2 ± 4.6	10.0 ± 7.6	57.6 ± 29.4
Married_(B)_	Yes (*n* = 40)	23.7 ± 4.0	9.9 ± 7.9	59.4 ± 30.7
	No (*n* = 61)	22.9 ± 4.1	9.9 ± 7.6	56.5 ± 31.3
First episode_(B)_	Yes (*n* = 75)	22.9 ± 4.1	9.8 ± 7.8	58.2 ± 30.4
	No (*n* = 26)	24.1 ± 3.9	10.2 ± 7.3	56.2 ± 33.1

HAMD = Hamilton Depression Rating Scale; IP = improvement percentage.

^a^ (B): Data recorded at baseline; (2Y): Data recorded at the two-year follow-up point.

^b^ There were no significant differences in data between patients according to categorical variables.

### Factors independently predicting the severity and IP of depression at follow-up

Table 4 shows the factors that independently predicted the HAMD_(2Y)_ score and the IP of the HAMD_(2Y)_ score. In the first model, DUD_(B)_, educational years_(B)_, and the HAMD_(B)_ score independently predicted the HAMD_(2Y)_ score. Among the three factors, DUD_(B)_ was the factor most strongly related to the HAMD_(2Y)_ score. In the second regression model, the DUD_(B)_ and educational years were significant factors that independently predicted the IP of the HAMD_(2Y)_ score.

**Table 4:** Independent variables predicting the severity and improvement percentage of depression at the two-year follow-up point ^a^^,^^b^.

Dependent variable	Independent variable	Beta	*t*	*P*
HAMD score_(2Y)_	age_(B)_	-0.17	-0.47	0.65
	gender_(B)_	0.10	1.04	0.30
	educational years_(B)_	-0.24	-2.22	0.03
	DUD_(B)_	0.32	2.54	0.01
	HAMD_(B)_	0.21	2.01	0.047
	HADS-A score_(B)_	-0.02	-0.16	0.88
	age at first MDE	0.21	0.57	0.57
	first MDE	0.05	0.20	0.84
	number of episodes	0.21	0.91	0.37
IP of the HAMD score_(2Y)_	age_(B)_	0.07	0.18	0.86
	gender_(B)_	-0.10	-0.93	0.36
	educational years_(B)_	0.23	2.08	0.04
	DUD_(B)_	-0.29	-2.15	0.03
	HAMD_(B)_	0.05	0.49	0.63
	HADS-A score_(B)_	-0.03	-0.22	0.83
	age at first MDE	-0.10	-0.25	0.80
	first MDE	-0.03	-0.12	0.91
	number of episodes	-0.20	-0.82	0.42

DUD = duration of untreated depression; MDE = major depressive episode; HADS-A = anxiety subscale of the Hospital Anxiety and Depression Scale; HAMD = Hamilton Depression Rating Scale; IP = improvement percentage.

^a^ (B): Data recorded at baseline; (2Y): Data recorded at the two-year follow-up point.

^b^ Multiple linear regressions with the forced entry method were used.

If the sample size was extended to include all subjects who accepted follow-up (n = 131, including 101 subjects without pharmacotherapy and 30 with pharmacotherapy at follow-up), the DUD_(B)_ remained a significant factor that predicted the HAMD_(2Y)_ score (beta = 0.28, t = 2.36, *p* = 0.02) and the IP of the HAMD_(2Y)_ (beta = -0.25, t = -2.06, *p* = 0.04) in the two regression models.

## Discussion

DUD_(B)_ was the most significant factor independently predicting the severity and IP of depression_(2Y)_ after controlling for the severities of depression_(B)_ and anxiety_(B)_, as well as demographic variables. DUD_(B)_ was significantly and positively correlated with the HAMD_(2Y)_ score, as well as significantly and negatively correlated with the IP of the HAMD_(2Y)_ score. These results showed that a longer DUD_(B)_ resulted in a greater severity of depression and a lower IP of depression at the two-year follow-up point, showing that DUD_(B)_ had a negative impact on the prognosis of depression. Our result was compatible with some previous studies, in which subjects were followed-up at points ranging from several months to one year [2]. However, our results were in contrast with those of a study by Furukawa et al., which showed that the severity of depression, but not DUD, was related to the outcome of depression at the two-year follow-up point [10]. Our study demonstrated that DUD_(B)_ remained a significant factor predicting the prognosis of depression at the two-year follow-up point, even though subjects had discontinued pharmacotherapy for nearly 1.5 years.

Three possible hypotheses might explain the negative impact of DUD_(B)_ on the prognosis of depression_(2Y)_. 1) MDD is associated with increased neuronal and glial cell death [16]. The grey matter volume in patients with MDD is negatively correlated with illness duration [17]. A longer untreated depression is associated with a reduction in the hippocampal volume, which is a neural marker for the scar effect of depression [18,19]. Antidepressant treatment is related to an increase in grey matter in the hippocampus in patients with depression [20,21]. Therefore, a longer DUD and delayed pharmacotherapy might be related to a greater severity of brain damage, leading to a poor prognosis of depression. 2) Depression causes functional impairment, which might further exacerbate the disorder. Improvement of depression earlier prevents the collapse of patients’ social support system or financial status. Patients with MDD who seek treatment in the early stages of depression might have a better knowledge of mental health. In fact, higher income and educational levels are associated with a higher remission rate [22]. Our study also demonstrated that educational years was significantly and negatively correlated with the severity of depression_(2Y)_. Therefore, a longer DUD implied a vicious cycle of depression that persists for a longer duration, which leads to a more difficult recovery. 3) In the natural course, self-recovery from depression without pharmacotherapy has a higher probability of occurring within the first 3 months of an episode [23]. MDD patients with a longer DUD might have more negative characteristics, which hinder the process of self-recovery, in terms of biological, psychological, and social dimensions. These negative characteristics might have negative impacts on treatment response and remission, as well as the long-term (two-year follow-up) prognosis of depression, even though subjects have discontinued pharmacotherapy.

Five points were worthy of note. 1) The severity of depression has been reported to be an important factor related to the prognosis of depression [24]. Our study demonstrated that DUD_(B)_ was more strongly associated with the severity and IP of depression_(2Y)_ than depression severity at baseline. In clinical practice, depression is evaluated using the scores of the 17 items of the HAMD. Clarification of DUD appeared to be simpler; therefore, compared with the HAMD, DUD is a more cost-effective measure by which to predict the prognosis of depression. 2) Our results demonstrated the possibility that early intervention by pharmacotherapy might be an important factor related to the prognosis of depression, even in patients who discontinue pharmacotherapy, because early intervention might disrupt the vicious cycle of depression. 3) Previous studies related to the impacts of DUE or DUI on the prognosis of depression often used treatment response or remission, which are categorical variables, as the major outcome indices [3,7,8]. Our study used the IP of the HAMD_(2Y)_ score, a continuous variable, as the outcome index, and a significant linear correlation between DUD_(B)_ and the IP of depression_(2Y)_ was noted. 4) Compared with the severity of depression at baseline, DUD_(B)_ was more strongly associated with the prognosis of depression. In clinical trials, DUD should be considered and controlled. 5) One study reported that the association of DUD with the outcome of depression post-pharmacotherapy was significant in patients suffering both a first or a recurrent episode [1]. This finding implied that there are no differences between the first episode and recurrent episodes in terms of the association of DUD with the outcome of depression at follow-up. Our results also demonstrated that the nature of the episode (i.e., first or recurrent) was not a significant factor related to the outcome of depression.

Several limitations or methodological issues should be emphasized. First, the results of this study only demonstrated the associations of the severity and IP of depression with DUD at the two-year follow-up point. The numbers of depressive episodes during the two years were not investigated. The severity and IP of depression at the two-year follow-up point might be unable to represent the longitudinal course of depression, because the course of depression might fluctuate. In future studies, the association of the longitudinal course of depression with DUD should be further investigated. Second, this clinical study was a neutral and observational study. Although the regression models included the total duration of pharmacotherapy and duration of discontinuance of pharmacotherapy as independent variables, the impacts of the two factors were unable to be fully excluded. Third, our results might have arisen from mixed effects of the above-described three possible hypotheses. Based on the first and second hypotheses, the vicious cycle of depression might be disrupted by early pharmacotherapy intervention. After discontinuation of pharmacotherapy, the course of depression might gradually return to a natural course, which is associated with the third hypothesis. The percentage contributions of the three hypotheses are unknown. Fourth, this study focused on MDD patients who had discontinued pharmacotherapy at the two-year follow-up point for two reasons: 1) most previous studies have focused on the impact of DUI or DUE on treatment response or outcome post-pharmacotherapy [2]. The impact of DUD on the outcome of depression among patients who discontinued pharmacotherapy has been neglected. 2) The content of pharmacotherapy at the two-year follow-up point was not controlled. Different kinds and varying dosages of medications might confound the severity of depression at follow-up. Fifth, restriction of prescription change in the first four weeks after enrollment might increase the risk of dropout due to side effects of medications.

In conclusion, a longer DUD was associated with a greater severity and lower IP of depression at follow-up. Early treatment might be an important factor related to the prognosis of depression. Our results further demonstrated that the impact of DUD on the prognosis of depression persisted even in subjects who had discontinued pharmacotherapy for nearly 1.5 years. Therefore, DUD is a valuable index for prediction of the long-term prognosis of depression and should be assessed in clinical practice.

## Supporting information

S1 Dataset The dataset includes demographic variables, duration of untreated episode, and scores of psychometric scales at baseline and 2-year follow-up. (XLS)
